# RNase MRP and the RNA processing cascade in the eukaryotic ancestor

**DOI:** 10.1186/1471-2148-7-S1-S13

**Published:** 2007-02-08

**Authors:** Michael D Woodhams, Peter F Stadler, David Penny, Lesley J Collins

**Affiliations:** 1 Allan Wilson Centre for Molecular Ecology and Evolution, Massey University, Palmerston North, New Zealand; 2 Bioinformatics Group, Department of Computer Science and Interdisciplinary Center for Bioinformatics, University of Leipzig, Härtelstraße 16-18, D-04107, Germany

## Abstract

**Background:**

Within eukaryotes there is a complex cascade of RNA-based macromolecules that process other RNA molecules, especially mRNA, tRNA and rRNA. An example is RNase MRP processing ribosomal RNA (rRNA) in ribosome biogenesis. One hypothesis is that this complexity was present early in eukaryotic evolution; an alternative is that an initial simpler network later gained complexity by gene duplication in lineages that led to animals, fungi and plants. Recently there has been a rapid increase in support for the complexity-early theory because the vast majority of these RNA-processing reactions are found throughout eukaryotes, and thus were likely to be present in the last common ancestor of living eukaryotes, herein called the Eukaryotic Ancestor.

**Results:**

We present an overview of the RNA processing cascade in the Eukaryotic Ancestor and investigate in particular, RNase MRP which was previously thought to have evolved later in eukaryotes due to its apparent limited distribution in fungi and animals and plants. Recent publications, as well as our own genomic searches, find previously unknown RNase MRP RNAs, indicating that RNase MRP has a wide distribution in eukaryotes. Combining secondary structure and promoter region analysis of RNAs for RNase MRP, along with analysis of the target substrate (rRNA), allows us to discuss this distribution in the light of eukaryotic evolution.

**Conclusion:**

We conclude that RNase MRP can now be placed in the RNA-processing cascade of the Eukaryotic Ancestor, highlighting the complexity of RNA-processing in early eukaryotes. Promoter analyses of MRP-RNA suggest that regulation of the critical processes of rRNA cleavage can vary, showing that even these key cellular processes (for which we expect high conservation) show some species-specific variability. We present our consensus MRP-RNA secondary structure as a useful model for further searches.

## Background

**Figure 1:**
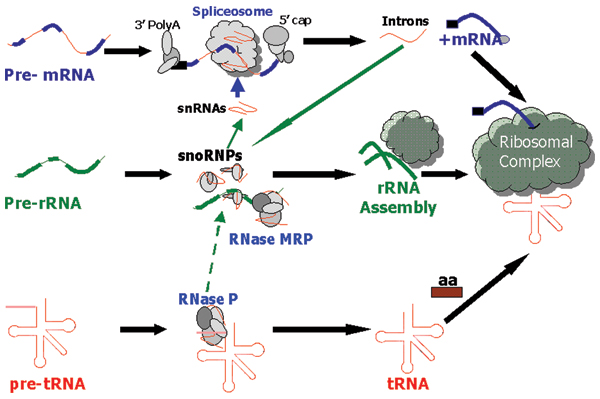
**The eukaryotic RNA-processing cascade. mRNA is cleaved by the spliceosome (comprised of snRNAs and proteins) to release the processed mRNA and introns**. Some introns contain snoRNAs which in turn modify snRNAs, tRNAs and rRNAs. RNase P (P) cleaves pre-tRNA while RNase MRP (MRP) cleaves rRNA. The ribosomal complex (comprised of rRNAs) brings the tRNAs and mature mRNAs together for translation. The involvement of RNase P in pre-rRNA processing has been questioned recently in [59].

There is high interest in discovering new roles of RNA in modern eukaryotes [1-4]. The number of putative ncRNAs (non-coding RNAs) in the mammals alone has increased about 20-fold in the last five years [1], thus any information on the origins and functions of well-established ncRNAs is relevant and timely. In eukaryotes a number of ncRNA-based molecules are directly involved in the cleavage and processing of other RNA molecules. A classic example is the cleavage of rRNA transcript by RNase MRP, a ribonucleoprotein complex consisting of a single RNA molecule and about 10 proteins [5-7]. The processing of RNA by RNA can extend through several layers such as the snRNAs (small nuclear RNAs) in the spliceosome release snoRNAs (small nucleolar RNAs) from introns which in turn are involved in the modification of rRNA, tRNA or snoRNAs (see Figure 1). The network of these processes is called the eukaryotic RNA-processing cascade [8]. This cascade centres on the processing mRNA, tRNA and rRNA and although each of these RNAs is cleaved in separate reactions, there are linkages between these reactions as shown in Figure 1. The question we ask here is: how ancient are these RNA-based processes?

Pre-mRNA contains introns that are processed by the spliceosome (consisting of 5 snRNAs and ~200 proteins) [9-11] but there is also further processing such as the addition of the 5'-cap and 3' poly-A-tail [12]. Although the capping and polyadenylation processes are not RNA-based reactions they do include some proteins found in the spliceosome [9]. The snRNAs within the spliceosomal complex not only direct the binding and coordination of the splice sites but are also implicated in the catalysis of the splicing reactions [13]. Some introns contain within them ncRNAs such as snoRNAs (involved in modification of rRNA, tRNA and snRNAs, reviewed in [14]) or miRNAs involved in the degradation of mRNA [15-17]. Similarly, pre-tRNA is processed by RNase P; a ribonucleoprotein consisting in eukaryotes of a single RNA and about 8–10 proteins [18]. RNase P (abbreviated here as P) is found throughout eukaryotes and prokaryotes [18,19], and thus may date back to the RNA-world [20,21]. Pre-rRNA is heavily processed and the A3 site in the ITS region is cleaved by the ribonucleoprotein RNase MRP (abbreviated here as MRP) generating the mature 5.8S rRNA [22-25].

MRP (Mitochondrial RNA Processing) was originally identified as an RNA-protein endoribonuclease that processes RNA primers for DNA replication in the mitochondria [26]. However, the majority of MRP (99%) is observed in the nucleolus where it is involved in pre-rRNA processing [18]. MRP probably has other essential functions [27] including roles in chromosomal segregation [28] and control of cell division [29]. Initial evolutionary studies (including [7]) only used MRPs from animals (13 mammals and frog), yeasts (20 Saccharomycetalian yeasts plus the fission yeast *S. pombe*) and plants (two dicotyledons). Although from the main multicellular groups, these sequences covered only a limited range within each group: land vertebrates within metazoans, Ascomycota within fungi, and the core eudicots within plants, leaving open the question whether MRP was present in the last common ancestor of eukaryotes [7].

**Figure 2:**
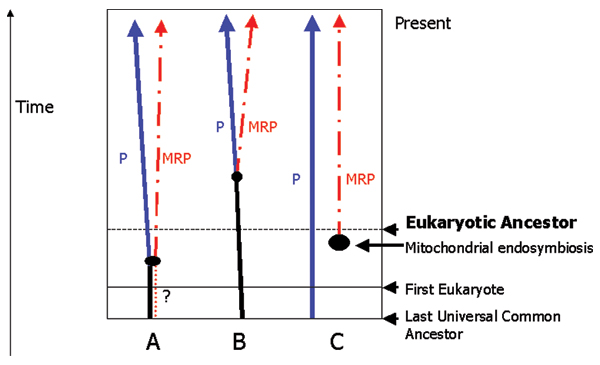
**Hypothesis for the origin of MRP**. This figure is based on [7], but modified to reflect present hypothesis of mitochondrial and eukaryotic evolution. The large black dots represent the point of duplication of the P-MRP ancestor. A: MRP was present in the last common ancestor of modern eukaryotes (the Eukaryotic Ancestor). Alternatively both MRP and P could have been present in the Last Universal Common Ancestor. B: MRP arose from a duplication of P after the Eukaryotic Ancestor, but before the ancestor of animals, fungi and plants. C: MRP arose from an early mitochondrial P within the Eukaryotic Ancestor.

We earlier [7] considered three hypotheses for the distribution of MRP (Figure 2). The first is that MRP is very ancient, occurring at least in the first eukaryotes. There are many variants on this model, and MRP could even be older in that most catalytic roles of RNA may derive from earlier stages in the origin of life, such as in the RNA-world [20]. Hypothesis B is that MRP arose from a duplication of P within modern eukaryotes (i.e. after the Eukaryotic Ancestor). This predicts a limited distribution of MRP in eukaryotes as well as explains the observation that P and MRP share most of their associated proteins [18]. Such duplication would be followed by specialization of the paralogous complexes, P being restricted to tRNA, and MRP to rRNA. There are several sub-hypotheses under this model, whether MRP took a new role in an internal excision in the rRNA precursor, or whether in eukaryotes, P initially carried out both reactions (with the precursors of tRNA and of rRNA) [7]. Hypothesis C is that MRP is derived from an early mitochondrial RNase P, followed by transfer of the gene to the nucleus, and co-option of MRP to a role in the nucleus in processing rRNA. Thus it is unclear whether the first role of MRP was in the nucleus and was later co-opted into a role in the mitochondrion, or vice versa.

In our earlier work it was concluded that the Hypothesis B was the most likely, that MRP had arisen within eukaryotes by a duplication of P, with subsequent specialization. The evidence against MRP coming from mitochondria (Hypothesis C) was that the secondary structure of MRP-RNA, as measured by RNA-shape metrics [7], was more similar to the eukaryote RNase P RNA than to the RNA from bacterial RNase P (the presumed source of the mitochondrial RNase P). Similarly, the apparent limited distribution of MRP in eukaryotes (at that time only in animals, fungi and plants) made it seem unlikely that MRP was present in the ancestral eukaryote. This left the duplication of P within eukaryotes (Hypothesis B) as the most likely. Two developments have changed our conclusions. Firstly, it now appears that the plant lineage and the fungi and animal lineage are widely separated on the eukaryote tree [30] and secondly, the recent characterisation of MRP in additional groups (as reported here and [31]) means that our initial conclusion must be reconsidered.

Piccinelli [31] recently extended the range of species from which MRP-RNA is characterised to include several protist species including apicomplexa. We have also used an MRP-search strategy to find candidate MRP-RNA sequences in other eukaryotic species. We have further examined MRP-RNA secondary structure and promoter regions from all known sequences to strengthen consensus models for MRP-RNA throughout eukaryotes. In the light of these results we discuss the presence of MRP in the last common ancestor of modern eukaryotes and re-examine its evolution and its relationship within the RNA-processing cascade throughout eukaryotic lineages. Because the deep divergences of eukaryotes is not known [30,32], our strategy has been to find candidate RNAs in as many lineages as possible, thus making our conclusions independent of the precise rooting of the eukaryotic tree [33].

## Results

### RNase MRP found throughout eukaryotes using specific search strategies

**Figure 3:**
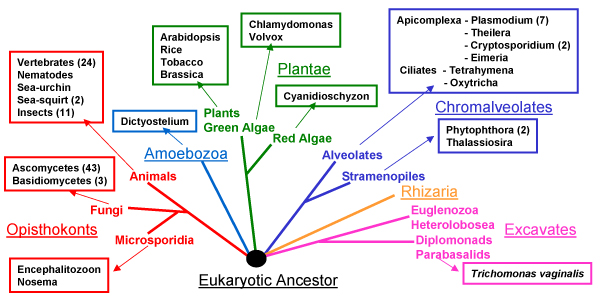
Distribution of MRP in eukaryotes. Only some sub-branches are given for each of the main groups. To date MRP has not yet been characterized from Giardia (Diplomonads), Trypanosoma (Euglenozoa), Entamoeba (Amoebozoa) and Caenorhabditis (Animals – nematodes). The general structure of the eukaryotic tree is based on [33].

MRP-RNA as a non-coding RNA (ncRNA) is not easy to identify in genomic sequences. Piccinelli [31] used a strategy based on hidden Markov Models (HMMs) of the P4 region of the MRP-RNA secondary structure to identify it in many eukaryotes. Our search strategy (in Materials and Methods) was also based on this P4 region and found MRP-RNA sequences from additional species. A eukaryotic tree (based on [32]) showing species from which MRP-RNA has been characterised is shown in Figure 3, and a full list of species is given in Additional File 1.

Our MRP-RNA candidates were checked against existing gene records and EST databases to support our bioinformatic approach. Of our new MRP-RNA candidates, two are found in EST databases: *D. melanogaster *[GenBank: CO153932] and *Plasmodium yoelii *[GenBank: BM161600 and GenBank: BM160961]. Validation of the MRP-RNA candidate from *Cryptosporidium parvum *with RT-PCR shows that this sequence is expressed in trophozoites (M. Irimia, data not shown). These Plasmodium and *C. parvum *results are particularly important because they show expression outside the animal, fungi and plant groups. In addition, five of our MRP-RNA sequences occur in Genbank records (excluding total sequence data): *G. gallus *[GenBank: AADN01006913], *T. nigroviridis *[GenBank: CAAE01012081], *P. falciparum *[GenBank: NC_004325] and *P. yoelii *[GenBank: AABL01002665]. That these do not overlap coding sequences is additional support for our computational approach. Interestingly, the candidate for the *D. melanogaster *MRP-RNA is on the negative strand of an intron in the Dmel_CG10365 gene [GenBank: AE014297], although the function of this gene is still unknown.

A single copy of the MRP-RNA gene was found in most species. However, the sea-urchin, *Strongylocentrotus purpuratus*, appears to have five closely related sequences, although some could turn out to be artefacts of the current assembly. Multiple MRP-RNA genes have been observed in plants [34]. Humans [35] typically have a single true copy and a number of pseudogenes and studies of the MRP in the pufferfish *Takifugu rubripes *[36] indicate only a single copy in this species.

**Figure 4:**
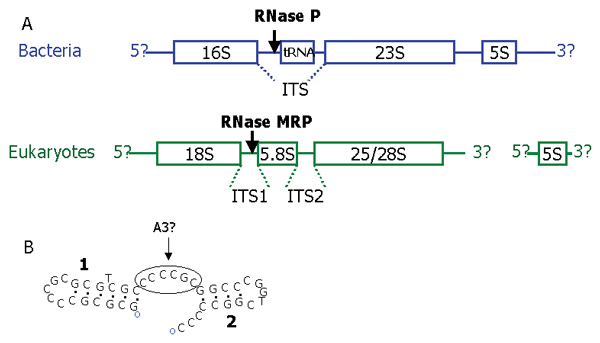
**Promoter regions of Human MRP, P and U6.A.** Organization of pre-rRNA based on [37]. In bacteria rRNA genes are co-transcribed as a polycistronic precursor (although exceptions are common). Most eukaryotes vary only in the length of their ITS regions, an extreme case being the microsporidian *Encephalitozoon cuniculi *which has completely lost its ITS2 having a fused 5.8S/28S subunit. RNase P and RNase MRP do not cleave the main transcripts but trim the ends of their respective substrates (the tRNA or 5.8S rRNA) after cleavage by other enzymes. In eukaryotes the 5S rRNA is transcribed separately by RNA polymerase III. **B. **The Diplomonad *Giardia lamblia *has the usual order of rRNA subunits with short ITS regions, however RNase MRP has not yet been characterized from this species. RNAstructure folding of G. lamblia ITS1 [56] showing a single stranded region between two stem loops that could possibly be an A3 site. Other foldings of this sequence and foldings of other sequences (DQ157272 and AF239841) produce just a single stem-loop.

As before [7,31] we did not find any MRP-RNA candidate in the Diplomonad *Giardia lamblia*. We examined the rRNA organisation in *G. lamblia *to determine if an A3 site, normally cleaved by MRP, was present. The order of rRNA subunits are in general similar throughout eukaryotes [37] (Figure 4A), though there is variation in the length and composition of ITS regions. Some microsporidia (e.g. *E. cuniculi *and *Nosema apis*) contain no ITS2 region and have no cleavage between the 5.8S and the 28S rRNA subunits. Another group of microsporidia, including *Nosema bombycis *and *N. spodopterae*, are exceptions to the standard eukaryote ordering, having the fused 5.8S/28S subunit before the 18S rRNA subunit [38]. *G. lamblia *rRNA has the standard ordering of eukaryotic rRNA, and its ITS1 region can be folded into a secondary structure with a short six nucleotide single stranded region between the two helices, a possible A3 cleavage site (Figure 4B). The ITS1 region from *Trichomonas vaginalis *(from which an MRP has been characterised) folds into a helix followed by an AC rich single-stranded region which could be the cleavage site for MRP. Future experimental analysis will determine the exact cleavage sites in these ITS regions.

The characterisation of MRP across a wide range of eukaryotes indicates that the evolutionary relationship between MRP and P is ancient, and that both MRP and P are likely to have been present in the last common ancestor of modern eukaryotes. Although seemingly obvious, this distribution analysis importantly places MRP in the RNA-processing cascade present in the Eukaryotic Ancestor and further allows us to examine other characteristics of MRP and its relationship to other processes present in this ancient cascade.

### Promoter analysis of candidate MRP-RNA sequences

With MRP the genes for proteins and for RNA are transcribed by different RNA polymerases; the protein genes by RNA Polymerase II, and the RNA by RNA Polymerase III (Type III promoter for U6 snRNA, 7SK and P-RNA; Type I for 5S rRNA,) [35,39,40]. RNA polymerase III transcription of MRP-RNA, P-RNA and U6 snRNAs has been characterised for some animal, plant and fungal species. Analysis of upstream regions of MRP-RNAs and the literature allowed us to analyse the promoter elements associated with RNA polymerase III transcription of MRP-RNA to determine if there was any conservation of its promoter elements.

**Figure 5:**
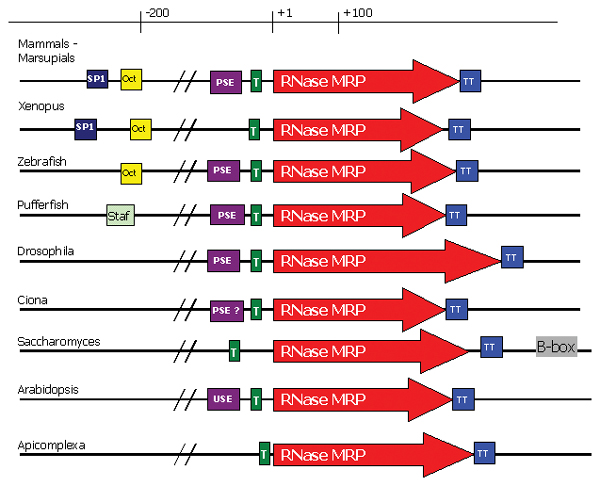
**MRP-RNA gene arrangement**. Genes transcribed by RNA polymerase III (type III) usually contain a PSE (proximal sequence element) consisting of a TATA signal and PSE motif, and a DSE (distal sequence element) consisting of either a SP1, Oct or Staf binding site. Distances shown are approximate only. Key: T – TATA signal; PSE/USE – ; Oct – Octamer binding site; SP1 – SP1 binding site; Staf – Staf binding site. ? – Possible site. TT – Poly T termination signal. B-box – Downstream B-box motif.

We find that MRP-RNA is probably transcribed by RNA polymerase III throughout eukaryotes but the set of RNA Polymerase III promoter elements may vary (see Figure 5). In general, vertebrate and plant MRP-RNA promoter regions contain an upstream TATA box, Proximal Sequence Element (PSE or USE) and a Distal Sequence Element (DSE) which can contain SP1, Staf and/or Octamer motifs [34,39-43]. In humans, the presence of the TATA box determines RNA polymerase specificity (i.e. RNA polymerase II or RNA polymerase III), with the other elements (e.g. PSE and DSE) enhancing transcription [44]. Plants require both the TATA box and the USE promoter (similar to the PSE element in vertebrates) with polymerase specificity determined by the spacing between the two elements [45]. In *Drosophila*, specificity is determined by the presence of the TATA box and the sequence of the PSE element [46]. However, the yeast *S. cerevisiae *uses a different RNA polymerase III promoter structure [44]. For example, the U6 snRNA promoter (similar to that expected in MRP-RNA) lacks PSE and DSE elements but instead includes a downstream B box ~120 nucleotides beyond the terminator [44].

Promoter comparisons show there can be differences in MRP-RNA promoter elements between some closely related species. For example in fish, the MRP-RNA promoter region for *Takifugu *previously described in [36] characterises a Staf promoter element (a binding site for the Staf transcriptional activator protein) in the DSE. We are unable to find any Staf-binding sequence in the other two fish MRP-RNAs. The Zebrafish and *T. nigroviridis *MRP-RNAs have potential SP1 binding sites, but as with the *Takifugu*, no Octamer sites could be determined. Mammals and chicken MRP-RNAs contain a similar arrangement of their MRP-RNA promoter elements. The frog MRP-RNA promoter regions have sequence motifs similar to mammals with a slightly different spacing between the elements within the DSE (the SP1 binding site is further upstream). Comparisons of six species of *Drosophila *(*D. melanogaster*, *D. pseudoobscura*, *D. yakuba*, *D. mojavensis*, *D. virilis *and *D. ananassae*) show a conserved PSE element (consensus sequence gcTTAtaATTCCCAAct) 23 nucleotides upstream of a TATA box (consensus sequence taaAta) which is about 16 nucleotides upstream of the transcription start site. The range of RNA polymerase III promoter structures and the lack of information about these promoter elements from protists make it difficult to identify promoter elements from protist MRP-RNA genes. Preliminary analyses of promoter regions from apicomplexa (*Plasmodium *and *Cryptosporidium*) indicate likely TATA boxes, but at this stage we cannot predict the presence of PSE or DSE elements. The AT richness of this region makes promoter prediction difficult until such time as we have more experimental information about promoters in apicomplexa. A table of promoter elements is available from the corresponding author upon request.

### Secondary structure analysis of MRP-RNA

**Figure 6:**
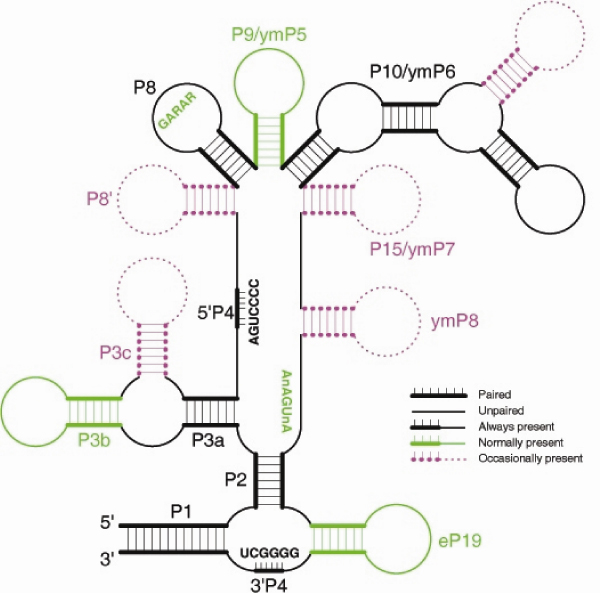
**Summary diagram of MRP-RNA secondary structure**. Black features (P1, P2, P3a, P4, P5, P7) are universally present. Green features are nearly universal, red features are observed in a few organisms of limited phylogenetic range. Thick lines are paired regions while unpaired regions are shown as thin lines. Conserved sequence motifs are indicated for the P4 (5' and 3') P8 and CRIV regions.

Analysis of the secondary structure of MRP-RNA (Fig. 6 and [25,31,47,48]) shows that the overall secondary structure is conserved throughout eukaryotes (Figure 6). Our naming of secondary structures in Figure 6 follows the convention that MRP-features are named after putatively homologous P-RNA features [25,49]. Features P1, P2, P3a, P4, P8 and P10 are found throughout all the MRP-RNA characterised to date while other features are nearly universal (P3b, P9 and eP19). A few features are observed in a limited phylogenetic range. Our consensus structure in Figure 6 is largely sequence independent, showing only the most conserved sequence motifs. This type of structure is essential for generating useful structure models for future computational searches for MRP-RNA.

Details of the consensus MRP-RNA secondary structure are as follows. P3 nearly universally has two helices (P3a, P3b) separated by an internal loop; except for the absence of P3b in microsporidia and an additional P3c helix in Cryptosporidia, *Dictyostelium discoideum*, *Anopheles *and the nematode *Brugia malayi*. The P10 helix is often long, typically 20–40 nucleotides, but in extreme cases (Pezizomycotina and Cryptosporidia) over 200 nucleotides. P15/ymP7 is present in Saccharomycetes yeasts and some apicomplexa. ymP8 is present in *S. pombe*, all Saccharomycetes with known MRP-RNAs, and some Pezizomycotina yeasts. The distinction between ymP7 and ymP8 is not always clear (e.g. *Coccidioides immitis*). There are some species-specific divergences from our secondary structure model. P19 is absent from *Ciona intestinalis *and P6 is absent from microsporidia and *D. discoideum*. P6 is absent from Cryptosporidia in previously published secondary structures [31], but these sequences can have an alternative folding which includes the P6 helix.

The GARAR motif (R = A or G), recently noted by [31] and discussed by [25] is present in most species to date and is a defining feature of the P8 helix. It is usually in a pentaloop with an occasional deletion to a tetraloop (GARA) [25]. The major variants of the motif are GARAR and GARA but others are possible including the fish *Tetraodon nigroviridis *(CAAAG), cabbage *Brassica oleracea *(GAGG), *Babesia bovis *(TAAAG) and *Eimeria tenella *(GCGAG). Cryptosporidia, Plasmodia, *T. vaginalis *and some ascomycete fungi do not appear to have the GARAR motif. The three basidiomycete fungi also have varied GARAR motifs; two species (*Coprinus cinereus *and *Laccaria bicolor*) have GAAAG as part of a bulge on P8. This region is suggested by [25] to be an MRP-specific region and thus will be important in the development of more MRP-specific search strategies. Another nearly universal motif is CR-IV (positioned 0–3 nucleotides before 3'P2). The sequence is AnAGUnA, the 'U' and the first 'A' sometimes being substituted. This motif is not recognisable in *T. vaginalis *and some Alveolata. In *S. purpuratus *(sea-urchin) the motif is in a non-standard position, overlapping and extending beyond P2.

## Discussion

The RNA cascade connects a number of RNA-based complexes where RNA is processing other RNA molecules. Figure 1 is a simplified model that shows the key processes and the main connections. Key processing complexes such as the spliceosome (snRNAs) [33], RNase P [19] and snoRNPs [50] are all seen as being ancestral to modern eukaryotes, even though details such as the intron recognition by the spliceosome [51] cannot yet be determined. The discovery of MRP across so many eukaryotes indicates that it was also part of this ancestral RNA-processing cascade. Given that MRP occurred so early in eukaryotes it is not surprising that MRP is now implicated in a number of other cellular processes (especially in well-studied species such as humans and yeast *S. cerevisiae*). As well as nuclear rRNA and mitochondrial primer cleavage functions, in *S. cerevisiae *at least, it has an additional function of promoting cell cycle progressing by cleaving CLB2 mRNA in its 5' UTR region at the end of mitosis [52,53]. It is possible that other functions of MRP may be found, especially when other RNA-processing systems are investigated.

One main conclusion from this study is that, with the placement of MRP in the RNA-processing cascade of the Eukaryotic Ancestor, we see little change in basic RNA-processing throughout eukaryotes. Eukaryotes and prokaryotes have basic differences in their processing of their rRNA transcripts [37]; the main eukaryotic transcript contains ITS1 (between the 18S and 5.8S) and ITS2 (between the 5.8S and 28S) whereas prokaryotes generally have only an ITS1 with the 5'end of the prokaryotic 23S with strong homology to the eukaryotic 5.8S sequence [54] (Figure 4A). Thus we find the 5.8S rRNA, either cleaved as a separate subunit, or fused to the large rRNA subunit (no ITS2 present). Typically within eukaryotes we find the 5.8S rRNA cleaved but not in prokaryotes. There are exceptions for both, microsporidia do not appear to have an ITS2 [55,56], and in prokaryotes RNase III cleaved IVS (intervening sequence) regions in α-proteobacteria have been found [54]. RNase III, which is involved in cleaving the prokaryotic rRNA transcript has now been implicated in ITS1 processing in *S. pombe *[57]. It is likely that the Eukaryotic Ancestor contained cleaved 5.8S rRNA, but we cannot yet determine if the last universal common ancestor (of eukaryotes and prokaryotes) contained a separate or fused 5.8S.

The cleavage of site A3 in the ITS1 region by MRP is similar to the cleavage of the tRNA in the bacterial system by P. However, we do not know whether the eukaryotic-type of RNA-processing cascade has evolved from the bacterial RNA-processing system. Bacteria, archaea and eukaryotes have probably changed their original RNA-processing cascade, each in their own evolutionary trajectory [58]. Nor do we know whether the ancestral P had either a simplified bacterial-like form (with a single protein), or had multiple proteins (like eukaryotes) which have been reduced in prokaryotes to a single protein. One piece of evidence for this second hypothesis is that the human Rpp29 (pop4) protein, shared by P and MRP, acts as a cofactor for the *E. coli *P-RNA [25]. We can no longer make the assumption that the prokaryotic models of RNase P and RNA-processing are ancestors of the eukaryotic complexes.

The high similarity of secondary structure between MRP and P [7] is indicative of an evolutionarily relationship, probably maintained by the sharing of numerous proteins between the MRP and P. However, it appears likely that P and MRP were already separate in the Eukaryotic Ancestor. The association of proteins with their respective MRP and P-RNAs may differ not only between P and MRP but may also vary between species [59]. Thus much of the large similarity in secondary structure between sections of MRP and P-RNAs (e.g. the P3-region indicated in [31]) is likely due to the constraints placed on the RNA molecules by their interactions with their common proteins even if some proteins interact transiently in some species.

The GARAG motif is an interesting addition to the MRP-RNA secondary structure. This pentaloop (or sometimes tetraloop) is potentially a protein or RNA binding site, which would explain its conserved nature. GNRA tetraloop motifs are also found in bacterial P (Type B but not Type B), archaeal P (both Type A and Type M) and also possibly in the ep9 helix of P from the yeast *S. cerevisiae *[25]. They are also common features of other ncRNAs. Identifying binding target sites for this motif in MRP may aid in understanding some of the differential protein binding [6]. The new consensus secondary structure model gives information required for future search strategies. Computational analysis of ncRNAs often use secondary structure and highly conserved sequence motifs rather than complete ncRNA sequences [7]. This model should allow more sophisticated MRP-RNA search algorithms to identify of MRP-RNA in additional eukaryotes.

The promoter region analysis indicates that RNA polymerase III is used throughout eukaryotes to transcribe MRP-RNA. It is interesting that there is such a range of promoter elements for MRP-RNA transcription and that the spacing between different elements appears critical in some species and not others. This may indicate that the regulation of MRP differs between groups of eukaryotes even for such an essential function as rRNA cleavage. There is little information about RNA polymerase III transcription in protists and the possible promoter regions are seldom reported when new ncRNAs are characterised.

Although MRP has been characterised in many eukaryotes, it has yet to be found in the nematodes *C. elegans *and *C. briggsae*, although complete genomes have been available for these species for several years. Similar exceptions are the protists *Giardia lamblia *and *Entamoeba histolytica. *However, MRP-RNA [31], was found in another nematode species *Brugia malayi *[31]. P-RNA has only recently been published for *C. elegans *[60] and *G. lamblia *[31,61] but even with all this new information we were still unable to find MRP-RNA in the above species. A recent survey for structured ncRNAs [62] based on comparative analysis of *C. elegans *and *C. briggsae *again did not result in a plausible MRP-RNA candidate. We have also not yet recovered any MRP-RNA from a *G. lamblia *RNA library (although we have recovered P-RNA) (S.X. Chen, data not shown). Nevertheless, in *C. elegans*, *G. lamblia *and *E. histolytica *the rRNA gene arrangement is generally the same as in other eukaryotes [63], although the ITS1 regions are very short in *G. lamblia *and *E. histolytica *[56]. Short ITS1 regions are also found in other species with reduced genomes such as *Trichomonas vaginalis *and the microsporidian *E. cuniculi *both of which have had MRP-rRNAs [31]. *G. lamblia *does not contain a nucleolus, but it is expected to contain a eukaryotic-like rRNA processing system due to the presence of many pre-rRNA processing proteins [64]. Some proteins that are usually shared between P and MRP have also been found in *G. lamblia *[65]. Two MRP-specific proteins (proteins not found in P, Smn1 and Rmp1) [66] have been characterised only in yeast and thus using MRP-specific proteins to indicate the presence of MRP, is not an option at this stage. The large evolutionary distance between Diplomonads and the only excavate from which MRP has previously been characterised (the Parabasalid, *Trichomonas vaginalis *[31]) means that MRP may be difficult to characterise in *G. lamblia *and even other excavates such as the Eugelenozoa (e.g. *Trypanosoma brucei*), using present techniques in computational analysis which rely heavily on sequence homology in the P4 region.

It is likely that the major protein and RNA components of the RNA processing cascade evolved before the Eukaryotic Ancestor (which is now seen to have come after the mitochondrial endosymbiosis [67]) It is interesting that MRP is still found in species that no longer contain mitochondria [31], but contain instead reduced organelles such as mitosomes or remnant mitochondria (apicomplexa and microsporidia) and hydrogenosomes (ciliates, parabasalids and some fungi) [67].

The RNA-processing cascade is now seen as a complex feature of the ancestral eukaryotic cell. Only when we understand which eukaryotic processes were in the Eukaryotic Ancestor we can then consider how they evolved.

## Conclusion

We present the organisation of RNA-processing in eukaryotes as a cascade of RNA-based processing reactions cleaving or modifying other RNA molecules. The main components of this cascade are conserved throughout eukaryotes and are likely to have been present in the Eukaryotic ancestor. We can now place MRP in this cascade and thus basic RNA-processing has been preserved in eukaryotes. Analysis of MRP-RNA promoter regions suggest, however, that regulation of these critical processes differs between species showing that even these key cellular processes are showing some species-specific variability. Computational searches for ncRNAs are difficult due to the necessary incorporation of secondary structure as well as sequence information. Our consensus secondary structure for MRP-RNA provides a useful model for further search strategies.

## Methods

### Searching genomes for RNase MRP RNA

The conserved regions around the P4 pseudoknot are important for finding potential candidate MRP-RNAs. A genome is scanned for regions similar to the conserved sequences from known MRP-RNAs then candidates are evaluated for stereotypical secondary structure. Candidates with suitable secondary structure are evaluated for upstream promoter regions expected for a gene transcribed by RNA polymerase III, then blasted against EST databases [68] for any indication that the candidate is expressed.

In more detail, the algorithm allows the search of genome sequences for closely located sequences which match or nearly match the consensus 5'P4 and 3'P4 regions (taken to be gaaAGuCCCC and acnnnanGGGGCUnannnu respectively, paired bases in uppercase). We have three levels of search criteria. Using the tightest criteria, 5'P4 and 3'P4 can be separated by 120 to 280 bases and we allow just one deviation from the consensus (either a single substitution of an unpaired base, or a substitution of a Watson-Crick pair by another such pair). The second, slightly looser, search criteria allows 100 to 360 bases between 5'P4 and 3'P4, and two deviations from the consensus. The loosest criteria allows a 80 to 500 base separation of P4, and two deviations from the consensus or a single violation of the expected Watson-Crick pairings. Genomes are scanned with the tightest criteria first, then with subsequent relaxed criteria if the first scan fails to identify an MRP candidate with suitable secondary structure. The Perl programs used are available upon request.

### Secondary structure analysis of MRP-RNA

Vertebrate [47] and yeast [39] secondary structures were obtained from the literature. For each new candidate sequence, we use RNAfold [69] and Mfold [70] to fold sequences of varying lengths prior to the 5'P4 region, to find a candidate P3 structure, and similarly the region prior to 3'P4 to find a candidate P9 structure. If successful, this identifies a small region to search for the P2 structure. Once these three structures are identified, the complete structure is easily obtained. If the number of candidates from the scanning stage is large, we use RNAmotif [61,71] to filter out candidates that do not have suitable P2 and P3 helices (RNAmotif descriptor files are available on request). Where we have MRP candidates from closely related species, we refine our structures by comparing sequences with ClustalX [72] and DIALIGN [73], and comparing structures using a range of RNA comparison software (Alifold from the Vienna RNA package [74], RNAforester [75], RNAshapes [76] and RNAcast [77]).

## Authors' contributions

MW carried out the search and secondary structure analysis and drafted the original manuscript. PFS contributed to the search of new and not easily available genomes. DP participated in the design of the study and contributed to the evolutionary discussions. LC carried out the promoter analysis and drafted the final manuscript. All authors read and approved the final manuscript.

## Supplementary Material

Additional file 1 MRP-RNA sequences from a wide distribution of eukaryotes. Click here for file
